# Supplementary material for: Global genome diversity of the Leishmania donovani complex
Source: eLife. 2020 Mar 25;9:e51243. doi: 10.7554/eLife.51243 (PMC7105377; doi:10.7554/eLife.51243)
Supplement: Transparent reporting form [file elife-51243-transrepform.docx]

***eLife’s* transparent reporting form**

We encourage authors to provide detailed information *within their submission* to facilitate the interpretation and replication of experiments. Authors can upload supporting documentation to indicate the use of appropriate reporting guidelines for health-related research (see [EQUATOR Network](http://www.equator-network.org/%20)), life science research (see the [BioSharing Information Resource](https://biosharing.org/)), or the [ARRIVE guidelines](http://www.plosbiology.org/article/info:doi/10.1371/journal.pbio.1000412) for reporting work involving animal research. Where applicable, authors should refer to any relevant reporting standards documents in this form.

If you have any questions, please consult our Journal Policies and/or contact us: [editorial@elifesciences.org](mailto:editorial@elifesciences.org).

**Sample-size estimation**

- You should state whether an appropriate sample size was computed when the study was being designed
- You should state the statistical method of sample size computation and any required assumptions
- If no explicit power analysis was used, you should describe how you decided what sample (replicate) size (number) to use

Please outline where this information can be found within the submission (e.g., sections or figure legends), or explain why this information doesn’t apply to your submission:

This manuscript describes the genome-wide diversity of globally distributed samples of the *L. donovani* complex. As such there is no main statistical method, for which power analysis was directly required.

Instead, for our choice of samples, we included and sequenced all isolate data from previous *Leishmania* strain collections available to us and spanning as much of the geographic distribution as possible: This included 97 strains we sequenced for the first time. Those strains had previously been isolated and were available to any of our co-authors at *Leishmania* strain collections.

Additionally, we included publicly available sequenced isolates of the *L. donovani* complex. As for those sequences corresponding genetic analysis was typically already available, we took subsamples of 2-3 isolates of each previously described genetic group per geographic region.

The relevant information can be found in the Material & Methods section under “Choice of samples & sample origin”)

**Replicates**

- You should report how often each experiment was performed
- You should include a definition of biological versus technical replication
- The data obtained should be provided and sufficient information should be provided to indicate the number of independent biological and/or technical replicates
- If you encountered any outliers, you should describe how these were handled
- Criteria for exclusion/inclusion of data should be clearly stated
- High-throughput sequence data should be uploaded before submission, with a private link for reviewers provided (these are available from both GEO and ArrayExpress)

Please outline where this information can be found within the submission (e.g., sections or figure legends), or explain why this information doesn’t apply to your submission:

As stated in the sample-size estimation statement, the main aspect of the project is to describe and highlight the genome-wide diversity of globally distributed natural isolates of parasites belonging to the *Leismania donovani* complex. Therefore, the focus is not per se testing for biological differences between “treatment groups” requiring replication.

However, in a few cases exploratory analysis tries to establish biological differences requiring replication:

This includes, the estimation of chromosome-specific magnitude of aneuploidy turnover. In this analysis, geographically and genetically distinct groups as identified in the analysis, were used as 4-7 biological replicates (Figure 2, Figure 2-figure supplement 2 and corresponding figure legends).

Similarly, the same 7 groups were used as biological replicates to test for a relationship between somy variability and sample heterozygosity (Figure 3 and corresponding figure legends).

Linkage disequilibrium (LD), was analysed for the 6 largest geographically and genetically distinct groups. Here, groups containing more than 7 isolates were additionally subsampled into 3 “pseudo-replicates” of group size 7. This was done to make LD analysis comparable between groups of varying size (Figure 5 and corresponding figure legends).

**Statistical reporting**

- Statistical analysis methods should be described and justified
- Raw data should be presented in figures whenever informative to do so (typically when N per group is less than 10)
- For each experiment, you should identify the statistical tests used, exact values of N, definitions of center, methods of multiple test correction, and dispersion and precision measures (e.g., mean, median, SD, SEM, confidence intervals; and, for the major substantive results, a measure of effect size (e.g., Pearson's r, Cohen's d)
- Report exact p-values wherever possible alongside the summary statistics and 95% confidence intervals. These should be reported for all key questions and not only when the p-value is less than 0.05.

Please outline where this information can be found within the submission (e.g., sections or figure legends), or explain why this information doesn’t apply to your submission:

The following statistical tests were performed in this manuscript:

Bootstrapping to get node support in the phylogeny:

Bootstrapping support was calculated by partitioning the genome in 10kb windows and for each of 1,000 bootstrap-replicates drawing windows with replacement. For each replicate distance matrices of Nei’s distances were calculated for the respective windows and used for phylogenetic reconstruction using neighbour-joining. Node support is provided in % as observed across bootstrap-replicates.

Relevant information can be found in the Material & Methods under section “Phylogenetic reconstruction”, Figure 1 and <https://github.com/susefranssen/Global_genome_diversity_Ldonovani_complex/blob/master/01_phylogenetic_reconstruction/A01_leish_donovaniComplex_StAMPP_window_bootstrap.R>.

Isolation-by-distance analysis:

The Mantel-test was performed to assess correlations between genetic and geographical pairwise distances between isolated strains. Statistical significance was evaluated based on 10,000 permutations.

Relevant information can be found in the Material & Methods under section “Population structure and IBD analysis”, Supplemental file 2 and <https://github.com/susefranssen/Global_genome_diversity_Ldonovani_complex/blob/master/02_IBD_analysis/a21_IBD_stats.r>.

Correlations of aneuploidy characteristics between groups:

Correlations were assessed for group-specific a) somy variability and b) mean somy of the 36 different chromosomes between the four largest groups using Spearman correlations and FDR as multiple testing correction.

Relevant information can be found in the Results under section “Aneuploidy”, figure 2, associated legend and <https://github.com/susefranssen/Global_genome_diversity_Ldonovani_complex/blob/master/04_aneuploidy/a07_somy_pop_subgroups.r>.

Relationship between somy variability and sample heterozygosity:

Linear regression was performed to model chromosome specific sample heterozygosity in response to somy variability. Tests were done for each previously identified phylogenetic group, followed by multiple testing correction using FDR.

Relevant information can be found in the Results under section “Heterozygosity”, figure 3, associated legend and <https://github.com/susefranssen/Global_genome_diversity_Ldonovani_complex/blob/master/04_aneuploidy/a07_somy_pop_subgroups.r>.

(For large datasets, or papers with a very large number of statistical tests, you may upload a single table file with tests, Ns, etc., with reference to sections in the manuscript.)

**Group allocation**

- Indicate how samples were allocated into experimental groups (in the case of clinical studies, please specify allocation to treatment method); if randomization was used, please also state if restricted randomization was applied
- Indicate if masking was used during group allocation, data collection and/or data analysis

Please outline where this information can be found within the submission (e.g., sections or figure legends), or explain why this information doesn’t apply to your submission:

This study looks at the genome-wide diversity of globally distributed samples spanning as much of the global distribution and genetic diversity described previously of the *Leishmania* parasite as possible. Therefore, there are no experimental groups in the classical way, e.g. to assess biological differences due to particular treatments.

However, phylogenetic analysis identified genetically diverged groups, which are subsequently investigated to describe differences between species and geographic regions.

Relevant information can be found in the Results under section “Evolution of the L*. donovani* complex” and figure 1.

**Additional data files (“source data”)**

- We encourage you to upload relevant additional data files, such as numerical data that are represented as a graph in a figure, or as a summary table
- Where provided, these should be in the most useful format, and they can be uploaded as “Source data” files linked to a main figure or table
- Include model definition files including the full list of parameters used
- Include code used for data analysis (e.g., R, MatLab)
- Avoid stating that data files are “available upon request”

Please indicate the figures or tables for which source data files have been provided:

The 97 samples sequenced for this study are deposited in ENA under the study accession numbers: ERP000767, ERP000966 and ERP009989 (https://www.ebi.ac.uk/ena/data/view). All metadata on the 151 isolates including ENA accession numbers of individual samples are summarized in Supplementary File 1 (see also https://microreact.org/project/_FWlYSTGf; Argimón et al., 2016). Summary statistics and annotations from this study are available in Supplementary Files 1 – 13. Analysis scripts generated and used in this study along with the corresponding data files are available on github <https://github.com/susefranssen/Global_genome_diversity_Ldonovani_complex>.

The code includes initial command line processing of the raw data as well as final analysis and plotting in R. The analysis in R can typically be relatively easily repeated by the reader when downloading the github repository as the code as well as the input data is provided to re-generate the majority of the analysis and figures in the manuscript.
